# Digital Prescription–Based Influenza Activity Forecasting in Jiangxi Province, China: Comparative Modeling Analysis Using Multisource Data

**DOI:** 10.2196/96131

**Published:** 2026-09-23

**Authors:** Rongrong Yang, Zilu Xu, Chi Zhang, Liping Qiu, Jie Qian, Jie Liu, Yonghai Dong, Zhili Zeng, Rui Shen, Luzhao Feng

**Affiliations:** 1 Department of HIV/AIDS and Sexually Transmitted Disease Control and Prevention, Ganzhou Center for Disease Control and Prevention, Ganzhou, Jiangxi, 341000, China; 2 School of Population Medicine and Public Health, Chinese Academy of Medical Sciences & Peking Union Medical College, Key Laboratory of Pathogen Infection Prevention and Control (Peking Union Medical College), Ministry of Education, State Key Laboratory of Respiratory Health and Multimorbidity; Public Health Emergency Management Innovation Center of Beijing Higher Education Innovation Center for Philosophy and Social Sciences, No. 9 Dongdansantiao, Dongcheng District, Beijing, 100730, China, 86 10 65120716; 3 School of Public Health, China Medical University, Shenyang, Liaoning, 110122, China; 4 Department of HIV/AIDS and Sexually Transmitted Disease Control and Prevention, Nanchang Center for Disease Control and Prevention, Nanchang, Jiangxi, 330009, China; 5 Scientific Research Affairs Section, Jiangxi Center for Disease Control and Prevention, Nanchang, Jiangxi, 330029, China; 6 Department of Surveillance Forecasting and Emergency Response, Jiangxi Center for Disease Control and Prevention, Nanchang, Jiangxi, 330029, China; 7 Department of Infectious and Endemic Disease Control and Prevention, Jiangxi Center for Disease Control and Prevention, Nanchang, Jiangxi, 330029, China

**Keywords:** influenza, digital prescriptions, early warning, forecasting, disease surveillance

## Abstract

**Background:**

Traditional laboratory-confirmed influenza surveillance involves a 1- to 2-week reporting delay and captures only patients who have sought care and received a diagnosis, limiting early warning. Digital prescription data have been shown to signal influenza activity early, but their predictive value for forecasting remains unclear.

**Objective:**

We aimed to evaluate the predictive value of digital prescription data for influenza forecasting by assessing lead times, contributions across forecasting horizons, and added value when combined with other real-time sources.

**Methods:**

Using daily data for Jiangxi Province, China (2022-2024), we characterized temporal relationships for 13 multisource indicators using prewhitened cross-correlation and performed Granger-based predictor screening restricted to the 2022 to 2023 development period. Incidence was then forecast 1 to 14 days ahead through expanding-window rolling-origin validation trained on 2022 to 2023 data and tested on 2024 data. Six models (autoregressive integrated moving average [ARIMA], seasonal ARIMA [SARIMA], Prophet, least absolute shrinkage and selection operator [LASSO], random forest, and explainable boosting machine) plus a persistence baseline were compared across 3 exogenous-input scenarios (no external input, digital prescriptions alone, and selected sources). Each indicator was also forecast from its own history and compared with observed incidence.

**Results:**

After prewhitening, the digital prescription rate led influenza incidence by 14 days, whereas the online search index lagged incidence by 1 day, indicating near-synchronous tracking. In the development period Granger analysis, digital prescription rate, online search index, and temperature showed significant predictor-to-incidence associations after false discovery rate (FDR) correction (FDR-adjusted *P*<.001, *P*=.003, and *P*=.008, respectively), whereas nitrogen dioxide showed a marginal association (FDR-adjusted *P*=.11) and was additionally included as an environmental covariate. At 1 day, performance was similar across models and input scenarios (*R*^2^=0.925‐0.961; persistence *R*^2^=0.948), but differences emerged at longer horizons. SARIMA performed best with the digital prescription rate as the sole exogenous input, and LASSO performed best with 4-source input, both reaching a mean *R*^2^ of 0.766 and root mean squared error (RMSE) of 0.240 across the 14 horizons. At 14 days, single-source SARIMA achieved an *R*^2^ of 0.510 (RMSE 0.318), and multisource LASSO achieved an *R*^2^ of 0.551 (RMSE 0.305), a gain of 0.064 in *R*^2^ and a 6.2% reduction in RMSE over LASSO without exogenous input. Forecasts of the digital prescription rate showed the closest temporal consistency with observed influenza incidence (mean *r*=0.897 vs 0.866 for the online search index).

**Conclusions:**

Digital prescriptions provide an early signal of provincial influenza activity, preceding routine reported incidence by approximately 2 weeks in this setting. Multisource input can further improve forecasting when paired with models capable of selecting informative signals, but indiscriminate integration does not necessarily help. This early signal may support earlier preparedness, including antiviral planning and health care resource allocation.

## Introduction

Influenza remains a major global public health challenge, causing 3 to 5 million severe cases and 290,000 to 650,000 respiratory deaths annually [1]. Accurate and timely forecasting is important for guiding public health preparedness, resource allocation, and early interventions [2]. However, traditional laboratory-confirmed sentinel surveillance involves a delay of 1 to 2 weeks between specimen collection and the availability of confirmed results in weekly reports [3]. Although notifiable disease reporting in China provides timelier daily case counts, reported incidence still reflects only cases that have completed symptom onset, facility-based care seeking, and clinical diagnosis and is weighted toward patients with more severe symptoms, whereas individuals with milder or early-stage illness may instead turn to online health services. Reported incidence may therefore lag underlying epidemic dynamics, limiting early warning.

In response to this limitation, digital data streams have emerged as complementary tools for influenza surveillance [4]. However, these sources capture different aspects of influenza-related behaviors and transmission processes and may have distinct limitations. Search trends provide timely information on health-related interests [5] but may reflect media-driven attention and other factors unrelated to disease activity [6], resulting in potential misalignment with epidemiological patterns [6]. Over-the-counter medication sales capture self-medication behavior [7], but they are not specific to influenza and may also reflect treatment for other respiratory conditions [8]. Mobility signals provide indirect information on population movement and transmission risk but require additional modeling assumptions [9]. Conventional pharmacy prescription data contain clinically authorized medication information; however, they typically depend on offline care pathways in which patients must visit health care facilities before obtaining medication, potentially introducing delays and selecting individuals who complete the full care-seeking process [10,11]. These limitations highlight the need for timely digital health data sources that capture clinician-mediated treatment-seeking behavior.

Digital prescription data generated through online health platforms provide a potential source for addressing these limitations [12,13]. Through online consultation, physician assessment, and prescription authorization, antiviral transactions represent treatment-seeking behavior recorded close to the time of clinical decision-making [13]. Unlike search activity, which primarily reflects information-seeking behavior and may be influenced by media attention or other external factors [6], digital prescription data reflect a subsequent health care action. In China, the expansion of digital health platforms and on-demand delivery systems for prescription antivirals, including oseltamivir and baloxavir marboxil, has generated transaction-level data that are available within 24 hours [14]. Previous studies have shown that digital prescription data capture population-level influenza dynamics and provide complementary information for influenza surveillance [14]. However, their value as an exogenous predictor for forecasting influenza incidence remains insufficiently quantified, including their temporal lead and incremental forecasting contribution and the added value of combining them with other real-time sources.

**Figure 1.**
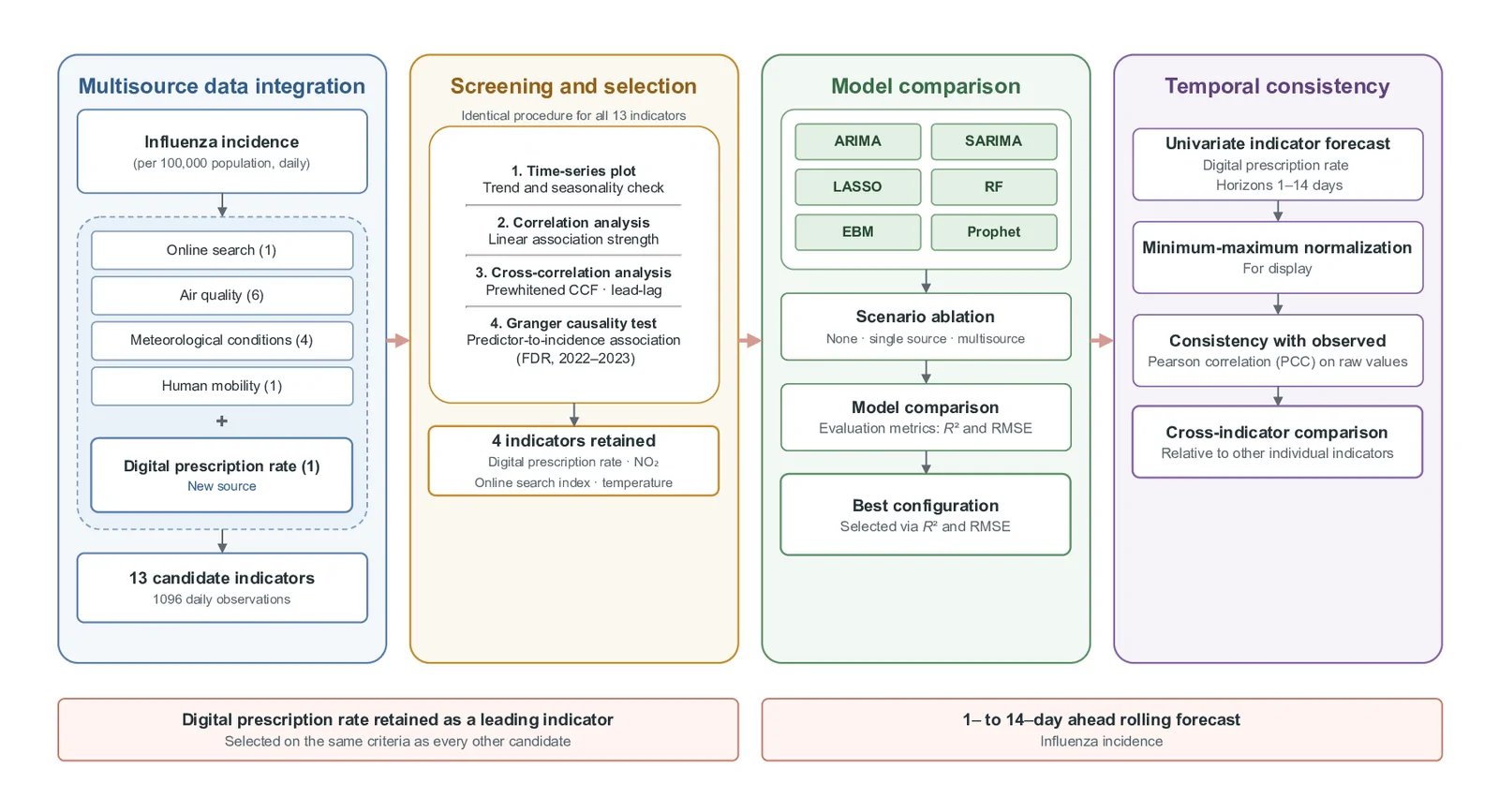
Study framework. The analysis proceeded in 4 stages. Thirteen candidate indicators from 5 data categories were assessed against daily influenza incidence. Lead-lag relationships were characterized over the full study period, whereas Granger screening was restricted to the 2022 to 2023 development period; 4 indicators were retained for forecasting. Six models were compared across 3 exogenous-input scenarios at horizons of 1 to 14 days. Temporal consistency between individual indicator forecasts and influenza incidence was also assessed. ARIMA: autoregressive integrated moving average; CCF: cross-correlation function; EBM: explainable boosting machine; FDR: false discovery rate; LASSO: least absolute shrinkage and selection operator; NO_2_: nitrogen dioxide; PCC: Pearson correlation coefficient; RF: random forest; RMSE: root mean squared error; SARIMA: seasonal ARIMA.

In this study, we evaluated digital prescription rate as an exogenous signal for provincial influenza forecasting in Jiangxi Province, China (Figure 1). We screened candidate indicators using a uniform time-series analysis framework, assessed their temporal relationships with influenza incidence, and compared forecasting performance under different exogenous-input scenarios across multiple models. We further compared the temporal consistency of the digital prescription rate relative to other individual indicators. Specifically, we aimed to determine (1) the lead time of digital prescription rate relative to influenza incidence, (2) whether digital prescription rate improves forecasting across different horizons, and (3) whether additional real-time sources provide incremental value under different modeling strategies.

## Methods

### Study Design and Data Sources

We carried out a comparative predictive modeling study spanning January 1, 2022, to December 31, 2024, integrating 6 daily data streams from Jiangxi Province (digital prescriptions, influenza surveillance, online search index, meteorological conditions, air quality, and human mobility) covering 1096 consecutive calendar days. To protect privacy, all data were aggregated at the provincial level, with all individual identifiers removed.

Digital prescription data were obtained from Meituan Healthcare, an online health platform that provides digital consultation and medication delivery services in China [14]. In this study, digital prescription data comprised transaction-level records for prescription-only antiviral medications, including oseltamivir and baloxavir marboxil. Digital prescription rate was defined as the daily number of unique purchasers of these medications per 100,000 residents in Jiangxi Province. Multiple transactions made by the same purchaser on the same day were counted once. Digital prescription date therefore represent the number of individuals obtaining prescribed antiviral medications rather than the number of prescriptions issued, filled, or delivered. Because these digital prescriptions were generated through an online health care pathway involving patient consultation, physician assessment, and prescription authorization, they reflect clinician-mediated treatment-seeking behavior but do not represent laboratory-confirmed influenza diagnoses [15].

Influenza incidence data were obtained from the China Information System for Disease Control and Prevention and expressed as the number of reported influenza cases per 100,000 population. As influenza is a statutory class C notifiable disease, cases are reported through the direct network reporting system within 24 hours of clinical diagnosis; daily counts indexed by report date are therefore available in near real time and reflect the values available at each forecast origin. Online search activity was obtained from the Baidu Index platform [16]. On the basis of our previous study [17], 8 Chinese-language search terms were predefined: “influenza,” “influenza A,” “influenza B,” “flu B,” “fever,” “sore throat,” “oseltamivir,” and “tamiflu.” Daily province-level search indexes for these terms were summed to construct a composite online search index representing overall influenza-related search activity. Human mobility data were obtained from the Baidu migration platform [18]. Daily migration rates were calculated following established methodology as follows: migration rate = (*M* × *OD* × coefficient/*P*) × 10^5^, where *M* is the migration scale index, *OD* is the directional flow proportion between provinces, and *P* is the population of the origin province; the coefficient is as described previously [19]. Missing values in the mobility series were imputed using dynamic harmonic regression with autoregressive integrated moving average (ARIMA) errors [20]. Meteorological variables, including temperature, relative humidity, wind speed, and precipitation, were obtained from the China Meteorological Data Service Center [21]. Air quality indicators, including PM_2.5_, PM_10_, carbon monoxide (CO), nitrogen dioxide (NO_2_), ozone (O_3_), and sulfur dioxide (SO_2_), were obtained from the China National Environmental Monitoring Centre [22]. All variables were aggregated at the provincial day level and aligned by calendar date.

### Indicator Screening and Selection

Thirteen candidate indicators from 5 categories, including digital prescription data, online search activity, human mobility, meteorological variables, and air quality indicators, were evaluated. Pearson correlation coefficients were first calculated to characterize concurrent associations between each indicator and influenza incidence. Raw cross-correlation functions were then calculated to describe unadjusted lead-lag relationships. To reduce correlations driven by autocorrelation and shared temporal patterns, weekly and annual seasonal patterns were first removed using Fourier terms, after which prewhitened cross-correlation analysis was also performed using the classic Box-Jenkins procedure [23]. For each indicator, an ARIMA model was fitted to the seasonally adjusted indicator series, and the same filter was applied to the seasonally adjusted incidence series before cross-correlations were calculated between the resulting residuals. Both raw and prewhitened cross-correlation functions were evaluated over lags ranging from –21 to +21 days, with negative lags signaling that the indicator preceded influenza incidence.

Granger tests were performed on the seasonally adjusted series to assess the temporal predictive relationships between each indicator and influenza incidence [24]. These analyses were restricted to the 2022 to 2023 development period to preserve the independence of the held-out 2024 test period. For each indicator, the lag order was selected using the Akaike information criterion, and each test examined whether past values of an indicator improved the prediction of subsequent influenza incidence beyond the information contained in the incidence series itself. *P* values from the concurrent correlation analyses and Granger tests were separately adjusted for multiple comparisons using the Benjamini-Hochberg false discovery rate (FDR) procedure. Granger associations were interpreted as predictive precedence rather than biological or mechanistic causation. Forecasting scenarios were constructed by integrating significant or marginal FDR-adjusted Granger evidence with prior epidemiological evidence. For indicators with marginal Granger evidence that were included in the multisource scenario based on prior epidemiological evidence, sensitivity analyses were conducted to assess their contribution to forecasting performance.

### Forecasting Framework and Analysis Methods

Influenza incidence was forecast at horizons of 1 to 14 days using an expanding-window rolling-origin validation framework. The initial models were trained using data from January 1, 2022, to December 31, 2023, and were evaluated during the held-out period from January 1, 2024, to December 31, 2024. Details for each model are provided in Table S1 in Multimedia Appendix 1. Predictor lags and other model inputs were constructed exclusively from information available at or before the forecast origin to prevent future information leakage.

ARIMA and seasonal ARIMA (SARIMA) were refitted at every forecast origin, whereas Prophet, least absolute shrinkage and selection operator (LASSO), random forest (RF), and explainable boosting machine (EBM) were refitted when a Page-Hinkley test detected drift in forecast errors or after a prespecified maximum interval. Three exogenous-input scenarios were compared: an autoregressive scenario without external indicators, a prescription-only scenario incorporating digital prescription rate as the sole exogenous predictor, and a multisource scenario incorporating digital prescription rate together with the other external indicators retained during screening. Exogenous predictors were aligned using a prespecified 14-day lag, which was applied consistently across models and input scenarios. This lag corresponds to the maximum forecast horizon, ensuring that all exogenous inputs were observed at or before each forecast origin for every 1– to 14–day ahead forecast and avoiding predictor-specific lag selection based on the observed data. Six forecasting approaches representing different statistical and machine learning strategies were evaluated: ARIMA, SARIMA, LASSO regression, Prophet, RF, and EBM. ARIMA and SARIMA represented classic time-series approaches, whereas Prophet used an additive time-series structure with linear growth and Fourier-based seasonal components. LASSO evaluated the linear contributions of multiple external predictors with variable selection, EBM modeled nonlinear additive effects, and RF allowed for both nonlinear relationships and interactions. Model-specific feature construction, preprocessing procedures, model updating strategies, hyperparameter settings, tuning strategies, and software packages are described in Table S1 in Multimedia Appendix 1 .

To evaluate the temporal consistency between individual indicators and influenza activity across forecast horizons, each retained indicator was also modeled separately using a univariate SARIMA model under the same expanding-window rolling-origin framework. Forecast indicator trajectories were compared with observed influenza incidence at the corresponding 1- to 14-day horizons using Pearson correlation coefficients. This analysis quantified the temporal consistency between each indicator’s forecast trajectory and subsequent influenza incidence.

### Model Evaluation

Forecasting performance was assessed using the coefficient of determination (*R*^2^) and root mean squared error (RMSE), calculated separately for each forecasting horizon across the target dates in the held-out period. Within each exogenous-input scenario, all models were evaluated on the same target dates and horizons. The persistence baseline carried the most recently observed influenza incidence value forward across each forecast horizon. Empirical coverage of the 95% prediction intervals was also evaluated. Analyses used Python (version 3.12; Python Software Foundation) and R (version 4.5.2; R Foundation for Statistical Computing).

### Ethical Considerations

This study was approved by the institutional ethics committee of the Chinese Academy of Medical Sciences and Peking Union Medical College (approval number CAMS&PUMC-IEC-2025-141). This study used only secondary data aggregated to the provincial level, from which all individual identifiers had been removed.

## Results

### Data Overview

The dataset comprised 1096 consecutive daily observations from Jiangxi Province, China, covering January 1, 2022, to December 31, 2024. The dataset was structured as a province-level daily time series, with influenza incidence as the outcome. The 13 candidate indicators were digital prescription rate, online search index, human migration rate, temperature, relative humidity, wind speed, precipitation, PM_2.5_, PM_10_, CO, NO_2_, O_3_, and SO_2_. The mean influenza incidence was 0.64 (SD 1.09) cases per 100,000 population. Before imputation, the mobility series was missing on 3% (n=33) of days; no other series had missing values. After imputation, all series were complete for subsequent analyses. Variable characteristics are reported in Table S2 in Multimedia Appendix 1.

### Indicator Screening and Temporal Relationships

Digital prescription rate showed a clear temporal lead while maintaining a strong concurrent association with influenza incidence. The online search index and digital prescription rate showed the strongest concurrent correlations with the incidence rate (*r*=0.80 and *r*=0.75, respectively; Table S2 and Figure S1 in Multimedia Appendix 1). The raw cross-correlation for the digital prescription rate was broad across the tested lags. After prewhitening, the digital prescription rate showed a distinct peak at a 14-day lead (*r*=0.19). The online search index had a slightly higher prewhitened peak (*r*=0.21) but lagged the reported incidence by 1 day, indicating near-synchronous tracking rather than a temporal lead (Table S3, Figure S2, and Figure S3 in Multimedia Appendix 1).

The 2022 to 2023 development period Granger analysis identified significant predictor-to-incidence associations for digital prescription rate (FDR-adjusted *P*<.001), online search index (FDR-adjusted *P*=.003), and temperature (FDR-adjusted *P*=.008; Table S3 and Figure S4 in Multimedia Appendix 1). NO_2_ showed a marginal predictor-to-incidence association (FDR-adjusted *P*=.11). Given this marginal association and prior epidemiological evidence linking ambient NO_2_ to influenza activity [25,26], NO_2_ was additionally included as an environmental predictor in the multisource scenario.

### Forecasting Performance and Added Value of Exogenous Inputs

**Figure 2.**
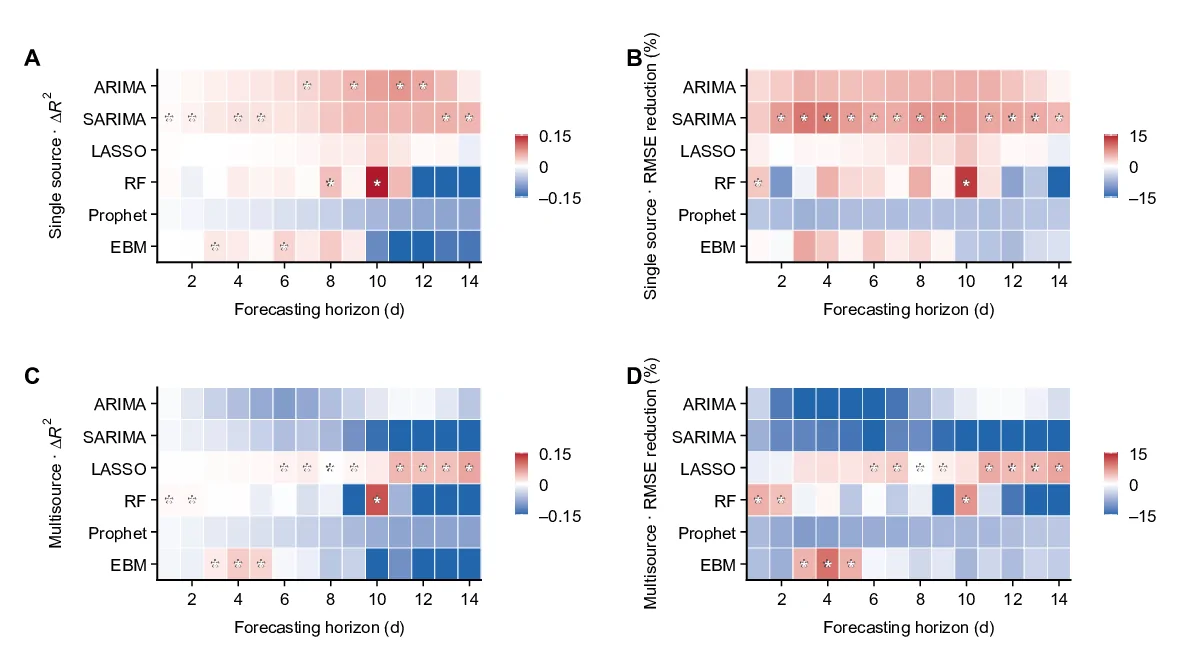
Incremental value of exogenous data over the no-exogenous baseline across forecasting horizons of 1 to 14 days. Panels A and B use a single exogenous source (digital prescription rate), whereas panels C and D use 4 sources (digital prescription rate, nitrogen dioxide, online search index, and temperature). Panels A and C show the absolute gain in *R*^2^ relative to each model’s own no-exogenous baseline, whereas panels B and D show the corresponding percentage reduction in root mean squared error (RMSE). Red indicates improved performance, blue indicates degraded performance, and white indicates no change; an asterisk marks the best-performing model at each horizon, not statistical significance . ARIMA: autoregressive integrated moving average; LASSO: least absolute shrinkage and selection operator; SARIMA: seasonal ARIMA.

The 2 leading exogenous-input configurations had the same rounded average performance and showed better point estimates than both the best no-exogenous model and the persistence baseline. Models were ranked within each input scenario by mean *R*^2^ across the 14 forecasting horizons. The best single-source configuration was SARIMA with digital prescription rate, whereas the best multisource configuration was LASSO with a 4-source input including digital prescription rate, online search index, temperature, and NO_2_. Both configurations had a mean *R*^2^ of 0.766 and a mean RMSE of 0.240 cases per 100,000 population. By comparison, the best no-exogenous configuration, LASSO, had a mean *R*^2^ of 0.748 and a mean RMSE of 0.248, whereas the persistence baseline performed less well, with corresponding values of 0.642 and 0.291 (Figure 2; Table S4 in Multimedia Appendix 1).

The contribution of external inputs became clearer at longer forecasting horizons, but the pattern differed by model. At 1 day, *R*^2^ ranged from 0.925 to 0.961 across models and input scenarios, and the persistence baseline achieved an *R*^2^ of 0.948. As the horizon increased, the digital prescription rate alone was most useful for SARIMA: the mean *R*^2^ increased from 0.733 to 0.766, and at 14 days, *R*^2^ increased from 0.458 to 0.510, whereas RMSE decreased from 0.334 to 0.318. Multisource input mainly benefited LASSO. Although the 3 LASSO configurations performed similarly at 1 day (*R*^2^=0.951‐0.954), multisource LASSO achieved an *R*^2^ of 0.551 and an RMSE of 0.305 at 14 days compared with 0.487 and 0.325 without exogenous inputs. This represented an increase in *R*^2^ of 0.064 and a 6.2% reduction in RMSE. To assess whether this improvement depended on the inclusion of NO_2_, we performed a sensitivity analysis excluding NO_2_. Mean *R*^2^ decreased from 0.766 to 0.752, and 14-day *R*^2^ decreased from 0.551 to 0.499 (Table S4 in Multimedia Appendix 1), indicating that NO_2_ contributed additional predictive information to LASSO in this setting. The contribution of external inputs remained model dependent. ARIMA showed a modest improvement with digital prescription rate alone, whereas external inputs generally reduced performance for Prophet, RF, and EBM; multisource input also reduced SARIMA performance (Table S4 in Multimedia Appendix 1).

**Figure 3.**
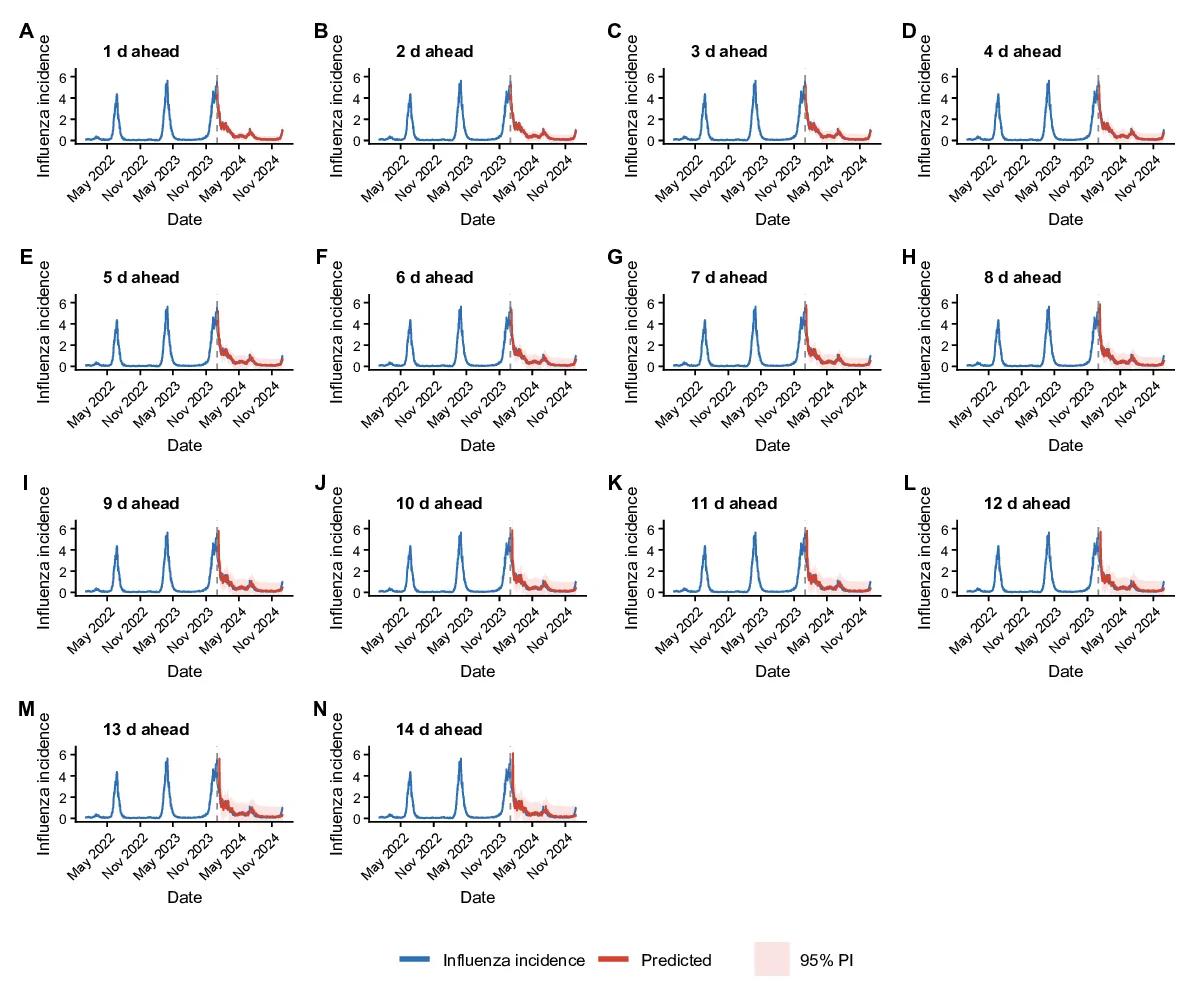
Predicted vs observed influenza incidence from the seasonal autoregressive integrated moving average model with a single exogenous source (digital prescription rate) at forecasting horizons of 1 to 14 days. Each panel (A-N) shows 1 horizon: the blue line represents observed influenza incidence, the red line represents the model prediction, and the shaded band represents the 95% prediction interval. The vertical dashed line marks the start of the held-out 2024 test period.

**Figure 4.**
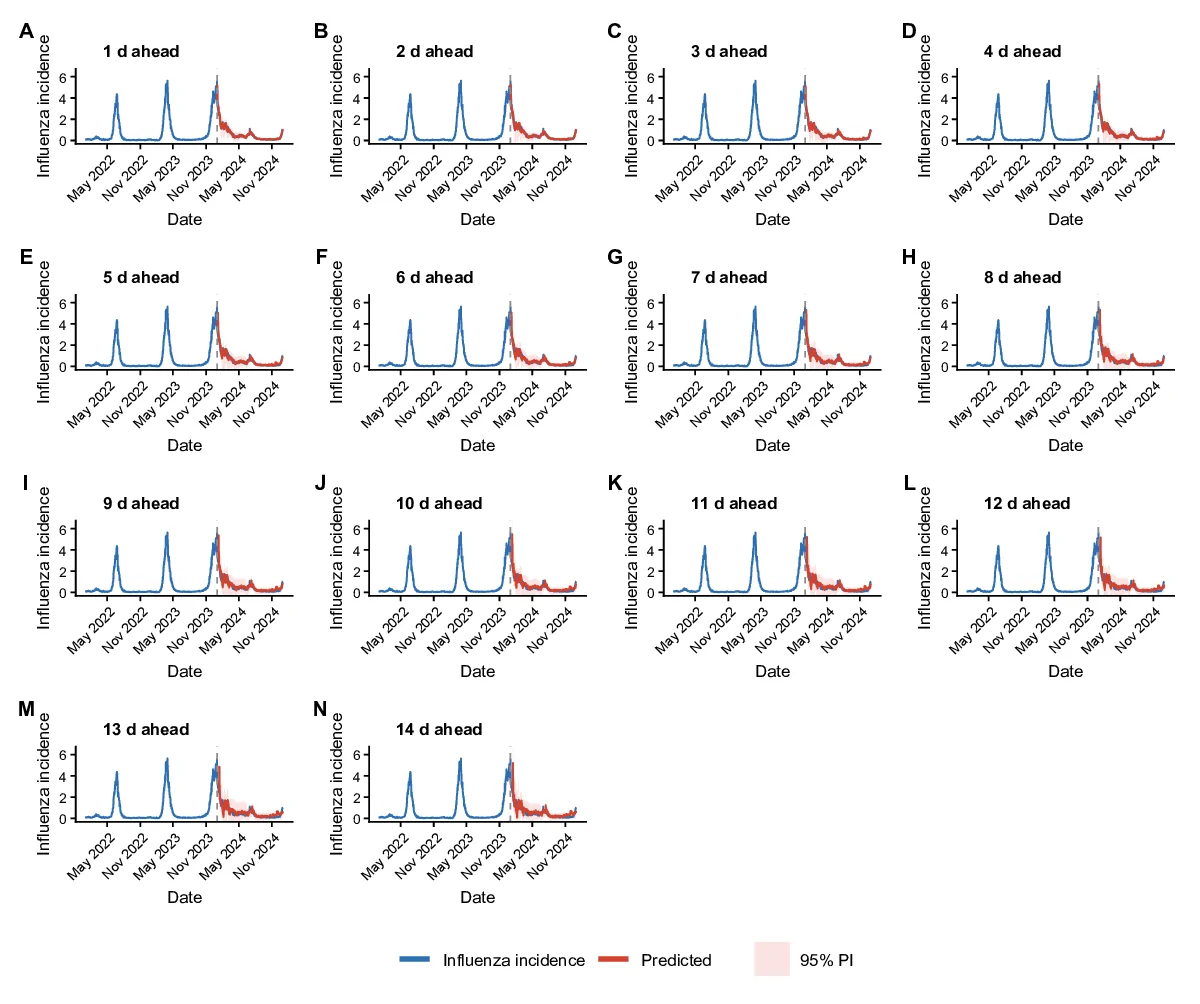
Predicted vs observed influenza incidence from the least absolute shrinkage and selection operator model with 4 exogenous sources (digital prescription rate, nitrogen dioxide, online search index, and temperature) at forecasting horizons of 1 to 14 days. Each panel (A-N) shows 1 horizon. The blue line represents observed influenza incidence, the red line represents the model prediction, and the shaded band represents the 95% prediction interval. The vertical dashed line marks the start of the held-out 2024 test period.

Because the main gains were concentrated in single-source SARIMA and multisource LASSO, forecast trajectories and uncertainty were examined for these 2 configurations. Both tracked the timing of the 2024 influenza wave more closely at shorter horizons, whereas predicted peaks became more attenuated and the 95% prediction intervals widened as the horizon increased. Across the 14 horizons, the empirical coverage of the 95% prediction intervals was 99.2% for single-source SARIMA and 92.8% for multisource LASSO (Figures 3 and 4).

### Temporal Consistency Between Indicator-Based Forecasts and Influenza Incidence

**Figure 5.**
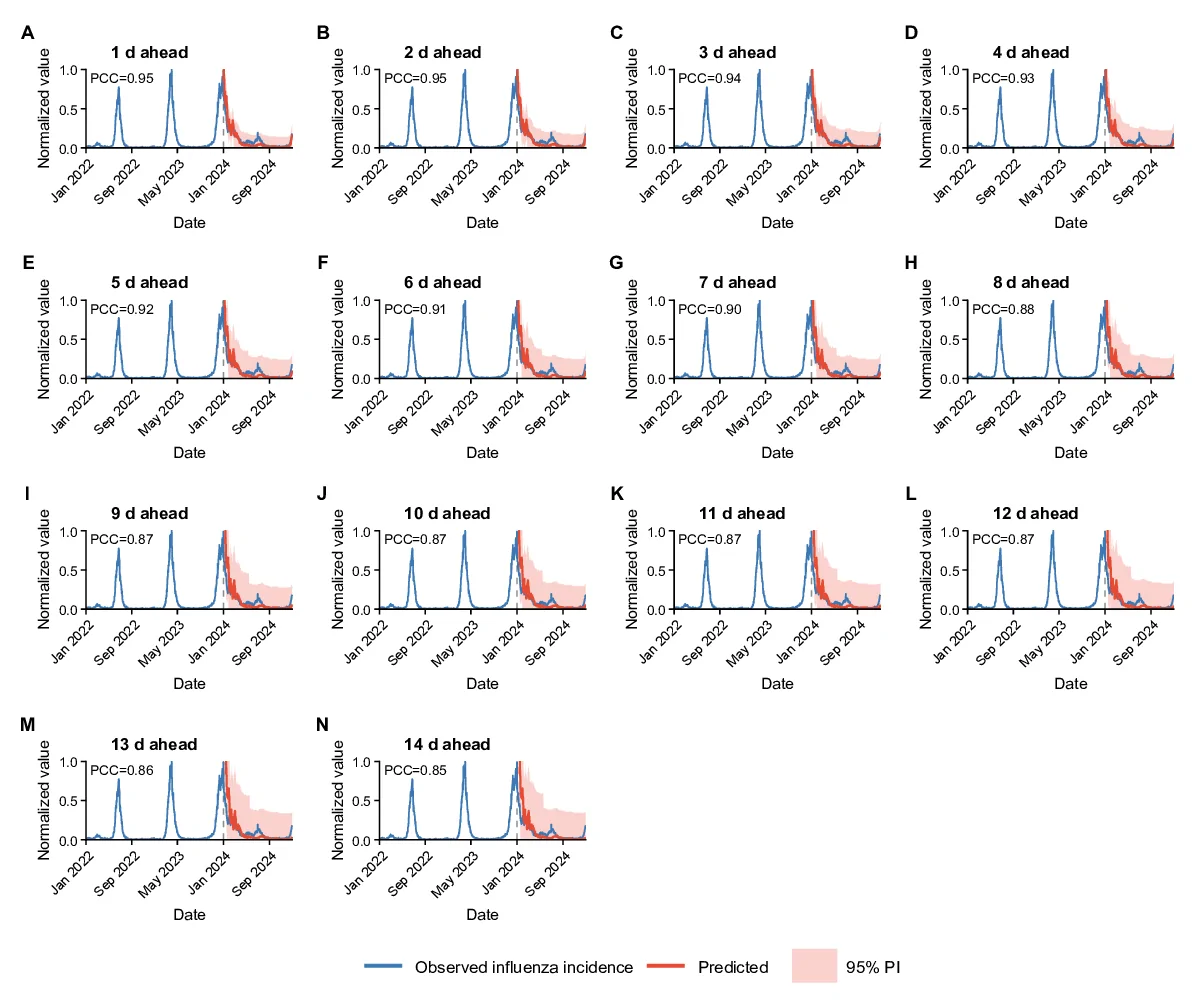
Temporal consistency between the forecast digital prescription rate and observed influenza incidence across horizons of 1 to 14 days. Each panel (A-N) shows 1 horizon. The red line represents the digital prescription rate forecast from a univariate seasonal autoregressive integrated moving average model, with the 95% prediction interval shaded; the blue line represents observed influenza incidence. The vertical dashed line marks the start of the 2024 out-of-sample period; both series were minimum-to-maximum normalized to (0, 1) for display, and the Pearson correlation coefficient annotated in each panel was computed on the raw, nonnormalized values.

Among the 4 indicators included in forecasting, forecasts of the digital prescription rate showed the closest and most consistent temporal agreement with subsequent influenza incidence. When each retained indicator was forecast based on its own history, the digital prescription rate showed the closest agreement with influenza incidence at every horizon (Figure 5). The correlation decreased from *r*=0.947 at 1 day to *r*=0.854 at 14 days, with a mean of 0.897 across the 14 horizons. The online search index ranked second, decreasing from *r*=0.897 to *r*=0.832, with a mean of 0.866. However, its near-synchronous cross-correlation indicated concurrent tracking rather than an earlier signal. Because the indicator and incidence series share seasonal structure, these correlations should be interpreted as temporal agreement and may partly reflect shared seasonality. Temperature and NO_2_ showed weaker agreement across all horizons (Figures S5-S7 in Multimedia Appendix 1).

## Discussion

### Principal Findings

Digital prescription data provided an early external signal of influenza activity in Jiangxi Province. After prewhitening, it led influenza incidence by approximately 2 weeks and showed a significant predictor-to-incidence Granger association in the 2022 to 2023 development period analysis. Forecasts of the digital prescription rate also showed the closest temporal consistency with subsequent influenza incidence across the 1- to 14-day horizons. These findings support digital prescription data as a potentially useful exogenous signal for influenza forecasting in this setting.

The value of external data depended on both the model and the forecasting horizon. Differences were small at 1 day, when recent incidence already provided a strong baseline. Incorporating digital prescription rate improved SARIMA performance, whereas the advantage of multisource input for LASSO became clearer at longer horizons. The best single-source and multisource configurations had the same rounded average performance, but adding several external sources did not consistently improve the other models. This suggests that multisource data are most useful when the model can retain informative signals and limit noise. These comparisons were conducted within a common rolling-origin framework using the same held-out dates and horizons and only information available at each forecast origin.

### Digital Prescription Data as an Early Signal

Digital prescription data differ from both online behavior and routine disease surveillance. Search activity mainly captures interest or concern [6], whereas digital prescriptions follow treatment seeking, clinical assessment, prescription authorization, and medication purchase [14]. Although these prescriptions do not necessarily represent laboratory-confirmed influenza diagnoses, they are closer to treatment demand than search activity or over-the-counter medication sales [14,27]. This may help explain why the digital prescription rate remained significantly associated with influenza incidence while also showing a clearer temporal lead.

The observed lead may reflect differences in when the two data streams are generated. Digital prescription data are generated when patients obtain prescribed antiviral medications, whereas reported influenza incidence reflects cases after health care seeking, clinical assessment, and reporting through the surveillance system. This interval should be interpreted as an empirical temporal relationship rather than a fixed biological lead or evidence that each digital prescription corresponded to a reported influenza case.

### Model-Dependent Value of External Inputs

The forecasting results favored a relatively simple use of the prescription signal. Digital prescription rate alone improved SARIMA performance and produced the same rounded mean performance as the best multisource configuration. A single clinically mediated data stream may therefore provide useful predictive information without the complexity of maintaining several external sources with different update schedules and data-generating processes.

The value of multisource input became more apparent at longer horizons. At short horizons, recent incidence already contained much of the information needed for prediction. As this autoregressive signal weakened, digital prescription rate and other external data sources, including online search index, temperature, and NO_2_, provided additional information related to treatment demand, epidemic conditions, and environmental context. The forecasting task included predictors with overlapping information across data sources and lag structures. By selecting informative variables and reducing the contribution of redundant predictors, LASSO may have provided a more stable and parsimonious model in this setting [28]. Although RF and EBM can capture nonlinear relationships, their additional flexibility did not result in improved performance in our dataset. These results indicate that the value of multisource input depends on how the forecasting model handles redundant or weak signals.

### Operational Implications for Layered Surveillance

These findings support a layered approach to influenza monitoring. Digital prescription data may provide an early indication of changing treatment demand, multisource models may add value at longer forecasting horizons, and routine surveillance remains necessary to confirm epidemic activity. Previous work has similarly shown that digital and traditional data streams can provide complementary information for influenza surveillance [29].

An additional warning interval of approximately 2 weeks could give health services more time to assess rising antiviral demand and plan medication supplies, staffing, and resource allocation [30]. Prediction intervals widened with the forecasting horizon, but empirical coverage remained high for the two leading configurations. These results describe forecasting performance rather than the effects of implementing the system in practice. Prospective studies are therefore needed to determine whether an earlier signal improves decisions concerning antiviral distribution, staffing, public health alerts, and other preparedness measures.

### Limitations

This study has some limitations. The analysis covered one province and used aggregated data, which may mask local variation and limit generalizability. Users of digital health platforms may also differ from the wider population [31]. Differences in digital health platform penetration, health care access, and health-seeking behavior across regions may influence prescription patterns and limit the direct transfer of the observed lead to other settings. The study period included changes in health behavior during and after the COVID-19 pandemic [32], and evaluation in a single held-out year cannot establish temporal stability.

In addition, linked symptom-onset dates, laboratory results, platform penetration rates, medication supply data, and promotional information were unavailable. Changes in physician prescribing practices, antiviral availability, public awareness, or platform use could affect digital prescription rate independently of influenza activity. These factors limit interpretation of the estimated lead and make it difficult to separate epidemiological changes from changes in platform use. The lead interval may vary across influenza seasons and surveillance settings because treatment-seeking behavior and reporting delays are not constant.

### Conclusions

Digital prescription data provided an early signal of provincial influenza activity, preceding routine reported incidence by approximately 2 weeks in this setting. Multisource input further improved forecasting when paired with a model capable of selecting informative signals, but adding external sources indiscriminately did not consistently improve performance. Incorporating this early signal may support earlier preparedness, including antiviral planning and health care resource allocation. Further validation across regions and influenza seasons is needed before routine implementation.

## Supplementary material

10.2196/96131 Multimedia Appendix 1 Supplementary tables and figures on model implementation, candidate indicator screening and temporal relationships, forecasting performance and sensitivity analyses, and temporal consistency with influenza incidence.

## Abbreviations

ARIMA: autoregressive integrated moving average
CO: carbon monoxide
EBM: explainable boosting machine
FDR: false discovery rate
LASSO: least absolute shrinkage and selection operator
NO_2_: nitrogen dioxide
O_3_: ozone
RF: random forest
RMSE: root mean squared error
SARIMA: seasonal autoregressive integrated moving average
SO_2_: sulfur dioxide
