# Supplementary material for: Digital Prescription–Based Influenza Activity Forecasting in Jiangxi Province, China: Comparative Modeling Analysis Using Multisource Data
Source: J Med Internet Res. 2026 Sep 23;28:e96131. doi: 10.2196/96131 (PMC13605229; doi:10.2196/96131)
Supplement: Supplementary file 1 [file jmir-v28-e96131-s001.docx]

Supplementary Tables and Figures

Contents

| Table S1. Model-specific implementation details for the influenza forecasting models | 2 |
| --- | --- |
| Table S2. Candidate indicator summary and contemporaneous Pearson correlations with influenza incidence | 4 |
| Table S3 Lead-lag analysis and development-period Granger screening of the 13 candidate indicators against influenza incidence | 5 |
| Table S4. Absolute forecasting performance of the six models under each exogenous data scenario, across horizons of 1 to 14 days | 6 |
| Figure S1. Time-series heatmap of influenza incidence and the 13 candidate indicators, January 1, 2022, to December 31, 2024 (1096 days) | 8 |
| Figure S2. Raw (unprewhitened) cross-correlation functions between each candidate indicator and influenza incidence | 9 |
| Figure S3. Prewhitened cross-correlation functions between each candidate indicator and influenza incidence | 10 |
| Figure S4. Granger test strength across daily lag orders for all 13 candidate indicators | 11 |
| Figure S5. Temporal consistency between the forecasted online search index and observed influenza incidence across horizons of 1 to 14 days. | 12 |
| Figure S6. Temporal consistency between the forecasted temperature and observed influenza incidence across horizons of 1 to 14 days. | 13 |
| Figure S7. Temporal consistency between the forecasted NO₂ and observed influenza incidence across horizons of 1 to 14 days. | 14 |

Table S1. Model-specific implementation details for the influenza forecasting models.

| Model | Forecasting strategy and feature construction^ab^ | Preprocessing and leakage control | Refitting strategy | Hyperparameters and tuning | Prediction intervals | Software package^c^ |
| --- | --- | --- | --- | --- | --- | --- |
| ARIMA | Path-based forecasting. Autoregressive structure was handled internally. The no-exogenous scenario used influenza incidence history alone; exogenous scenarios entered the scenario-specific inputs as regressors. | No feature scaling. Exogenous inputs were fixed at a 14-day lag and were dated on or before the forecast origin. | Re-estimated at every forecast origin. Model order was reidentified when residual diagnostics indicated inadequate whiteness. | Orders selected with auto.arima using BIC and stepwise search; seasonal terms disabled. No k-fold cross-validation. | Native model-based 95% prediction intervals. | forecast (R) |
| SARIMA | Path-based forecasting with internal autoregressive and seasonal structure. Scenario-specific external inputs were supplied as regressors. | No feature scaling. Exogenous inputs were fixed at a 14-day lag and were dated on or before the forecast origin. | Re-estimated at every forecast origin. Model order was reidentified when residual diagnostics indicated inadequate whiteness. | Orders selected with auto.arima using BIC and stepwise search; weekly seasonal period fixed at 7 days. No k-fold cross-validation. | Native model-based 95% prediction intervals. | forecast (R) |
| Prophet | Path-based forecasting with linear growth, weekly seasonality, yearly seasonality, influenza incidence lags of 1-14 days, and scenario-specific exogenous inputs as additional regressors. | No manual standardization. When an autoregressive regressor would otherwise refer to a post-origin observation, the last observed influenza incidence value was carried forward. | Refitted when a Page-Hinkley detector signaled forecast-error drift, subject to minimum and maximum refit intervals specified in the analysis code. | Linear growth; weekly seasonality; yearly seasonality with Fourier order 8. Other settings retained at package defaults; no separate grid search or k-fold cross-validation. | Native model-based 95% prediction intervals. | prophet (R) |
| LASSO | Direct, nonrecursive multistep forecasting with a separate model for each horizon h. Features comprised influenza incidence values from day T-14 through T; exogenous predictors were aligned at a 14-day lag relative to the forecast target date. | Predictors were standardized internally by glmnet at each model fit. All features were dated on or before forecast origin T. | Refitted when a Page-Hinkley detector signaled forecast-error drift, subject to minimum and maximum refit intervals specified in the analysis code. | L1 penalty with α=1. λ selected by BIC along the default glmnet regularization path. No k-fold cross-validation. | 95% intervals were estimated using empirical residual quantiles from recent out-of-sample forecast errors; in-sample residuals were used when insufficient forecast errors were available. | glmnet (R) |

Table S1 (continued). Model-specific implementation details for the influenza forecasting models.

| Model | Forecasting strategy and feature construction^ab^ | Preprocessing and leakage control | Refitting strategy | Hyperparameters and tuning | Prediction intervals | Software package^c^ |
| --- | --- | --- | --- | --- | --- | --- |
| RF | Direct, nonrecursive multistep forecasting with a separate model for each horizon, using the same origin-anchored feature set as LASSO. | No feature standardization. All autoregressive and exogenous predictors were available at or before the forecast origin. | Refitted when a Page-Hinkley detector signaled forecast-error drift, subject to minimum and maximum refit intervals specified in the analysis code. | 400 trees; minimum node size 5; impurity-based variable importance. mtry tuned over floor(sqrt(p)), floor(p/3), and floor(p/2) using out-of-bag error, where p is the number of predictors. | 95% intervals were estimated using empirical residual quantiles from recent out-of-sample forecast errors; in-sample residuals were used when insufficient forecast errors were available. | ranger (R) |
| EBM | Direct, nonrecursive multistep forecasting with a separate model for each horizon, using the same origin-anchored feature set as LASSO and random forest. | No manual standardization. All autoregressive and exogenous predictors were available at or before the forecast origin. | Refitted when a Page-Hinkley detector signaled forecast-error drift, subject to minimum and maximum refit intervals specified in the analysis code. | Purely additive model; pairwise interactions disabled; 8 outer bagging rounds. Other settings retained at package defaults. | 95% intervals were estimated using empirical residual quantiles from recent out-of-sample forecast errors; in-sample residuals were used when insufficient forecast errors were available. | interpret (Python) |
| Persistence baseline | The most recently observed influenza incidence at forecast origin T was carried forward as the prediction for every horizon. | No preprocessing. | No model fitting or refitting. | No hyperparameter tuning. | Not applicable. | Custom implementation (R) |

^a^No-exogenous input included influenza incidence history alone; single-source input added digital prescription rate; multisource input added digital prescription rate, NO₂, online search index, and temperature. The same scenario definitions were used for all six models.

^b^Models were trained initially on January 1, 2022, through December 31, 2023, and evaluated on the held-out 2024 period using an expanding-window rolling origin advanced one day at a time. Forecast horizons ranged from 1 to 14 days.

^c^All stochastic components used seed 42. Analyses were conducted using R 4.5.2 and Python 3.12.

ARIMA: autoregressive integrated moving average; BIC: Bayesian information criterion; EBM: explainable boosting machine; LASSO: least absolute shrinkage and selection operator; RF: random forest; SARIMA: seasonal ARIMA.

Table S2. Candidate indicator summary and contemporaneous Pearson correlations with influenza incidence.

| Variable | Category | Unit | Source | Pearson *r*^a^ | Pearson *P*^b^ (FDR) | *N*^c^ |
| --- | --- | --- | --- | --- | --- | --- |
| Influenza incidence | Outcome | per 100,000 | CISDCP | — | — | 1096 |
| Online search index | Online search | index (unitless) | Baidu Index | 0.80 | <.001 | 1096 |
| Digital prescription rate | Digital prescription data | per 100,000 population | Meituan Health | 0.75 | <.001 | 1096 |
| NO_2_ | Air quality | µg/m³ | CNEMC | 0.25 | <.001 | 1096 |
| O_3_ | Air quality | µg/m³ | CNEMC | −0.22 | <.001 | 1096 |
| Temperature | Meteorological condition | °C | CMDC | −0.21 | <.001 | 1096 |
| CO | Air quality | mg/m³ | CNEMC | 0.20 | <.001 | 1096 |
| SO_2_ | Air quality | µg/m³ | CNEMC | 0.17 | <.001 | 1096 |
| PM_10_ | Air quality | µg/m³ | CNEMC | 0.17 | <.001 | 1096 |
| PM_2.5_ | Air quality | µg/m³ | CNEMC | 0.14 | <.001 | 1096 |
| Relative humidity | Meteorological condition | % | CMDC | 0.06 | .048 | 1096 |
| Precipitation | Meteorological condition | mm | CMDC | 0.03 | .430 | 1096 |
| Wind speed | Meteorological condition | m/s | CMDC | −0.01 | .880 | 1096 |
| Migration rate | Human mobility | per 100,000 | Baidu Migration | 0.00 | .880 | 1096 |

^a^Contemporaneous Pearson correlation with influenza incidence. Indicators are ordered by absolute *r*.

^b^Two-sided *P* values adjusted for 13 comparisons using the Benjamini-Hochberg procedure.

^c^Number of observations entering the analysis. The migration rate includes 33 imputed days (see Methods); all other series were fully observed.

CISDCP: China Information System for Disease Control and Prevention; CMDC: China Meteorological Data Service Center; CNEMC: China National Environmental Monitoring Centre; FDR: false discovery rate; r: Pearson correlation coefficient.

**Table S3.** Lead-lag analysis and development-period Granger screening of the 13 candidate indicators against influenza incidence

| Variable | Prewhitened CCF peak, *r*^a^ | Prewhitened  Lead, days^b^ | Prewhitened CCF *P*^c^ | Predictor-to-incidence Granger, FDR-adjusted *P*^d^ |
| --- | --- | --- | --- | --- |
| Digital prescription rate | 0.19 | 14 | <.001 | <.001 |
| Online search index | 0.21 | −1 | <.001 | .003 |
| Temperature | 0.12 | 18 | <.001 | .008 |
| NO_2_ | 0.08 | −16 | .005 | .11 |
| SO_2_ | 0.11 | 16 | .001 | >.10 |
| PM_2.5_ | 0.10 | −21 | <.001 | >.10 |
| PM_10_ | 0.10 | −21 | .002 | >.10 |
| Migration rate | 0.08 | −5 | .009 | >.10 |
| CO | 0.07 | −1 | .023 | >.10 |
| Relative humidity | −0.06 | −4 | .042 | >.10 |
| Wind speed | −0.06 | 19 | .061 | >.10 |
| O_3_ | 0.06 | −4 | .064 | >.10 |
| Precipitation | 0.04 | 3 | .232 | >.10 |

^a^ Largest absolute prewhitened cross-correlation over lags of ±21 days, computed over the full study period (2022–2024).

^b^ Number of days by which the indicator precedes influenza incidence; negative values denote lagging.

^c^ Two-sided *P* value for the prewhitened CCF peak assessed against the Bartlett large-lag standard error, computed over the full study period (2022–2024).

^d^ Predictor-to-incidence Granger tests restricted to the development period (2022–2023); *P* values adjusted by the Benjamini-Hochberg false discovery rate procedure.

CCF: cross-correlation functions.

Table S4. Absolute forecasting performance of the six models under each exogenous data scenario, across horizons of 1 to 14 days.

| Scenario^a^ | Model | *R*^2b^  (mean, h1–h14) | RMSE  (mean, h1–h14) | *R*^2^ (h1) | *R*^2^  (h14) | RMSE  (h14) |
| --- | --- | --- | --- | --- | --- | --- |
| Baseline | Persistence | 0.642 | 0.291 | 0.948 | 0.200 | 0.406 |
| No exogenous | LASSO | 0.748 | 0.248 | 0.953 | 0.487 | 0.325 |
|  | SARIMA | 0.733 | 0.257 | 0.952 | 0.458 | 0.334 |
|  | Prophet | 0.623 | 0.297 | 0.949 | 0.150 | 0.419 |
|  | RF | 0.599 | 0.401 | 0.956 | 0.030 | 0.687 |
|  | ARIMA | 0.586 | 0.312 | 0.945 | 0.128 | 0.424 |
|  | EBM | 0.496 | 0.457 | 0.934 | −0.228 | 0.773 |
| Single source | SARIMA | 0.766 | 0.240 | 0.955 | 0.510 | 0.318 |
|  | LASSO | 0.754 | 0.245 | 0.954 | 0.472 | 0.330 |
|  | ARIMA | 0.619 | 0.299 | 0.948 | 0.142 | 0.421 |
|  | Prophet | 0.578 | 0.315 | 0.944 | 0.062 | 0.440 |
|  | RF | 0.572 | 0.407 | 0.959 | −0.401 | 0.825 |
|  | EBM | 0.473 | 0.464 | 0.934 | −0.275 | 0.787 |

Table S4 (continued). Absolute forecasting performance of the six models under each exogenous data scenario, across horizons of 1 to 14 days.

| Scenario^a^ | Model | *R*^2b^  (mean, h1–h14) | RMSE  (mean, h1–h14) | *R*^2^ (h1) | *R*^2^  (h14) | RMSE  (h14) |
| --- | --- | --- | --- | --- | --- | --- |
| Multisource (without NO_2_) | LASSO | 0.752 | 0.247 | 0.953 | 0.499 | 0.321 |
|  | SARIMA | 0.621 | 0.301 | 0.945 | 0.175 | 0.412 |
|  | Prophet | 0.575 | 0.317 | 0.943 | 0.065 | 0.439 |
|  | ARIMA | 0.545 | 0.334 | 0.941 | 0.087 | 0.434 |
|  | RF | 0.514 | 0.429 | 0.958 | −0.561 | 0.871 |
|  | EBM | 0.489 | 0.459 | 0.928 | −0.204 | 0.765 |
| Multisource (with NO_2_) | LASSO | 0.766 | 0.240 | 0.951 | 0.551 | 0.305 |
|  | SARIMA | 0.624 | 0.300 | 0.944 | 0.171 | 0.414 |
|  | Prophet | 0.572 | 0.318 | 0.942 | 0.061 | 0.440 |
|  | ARIMA | 0.544 | 0.335 | 0.941 | 0.076 | 0.437 |
|  | RF | 0.542 | 0.418 | 0.961 | −0.451 | 0.840 |
|  | EBM | 0.493 | 0.457 | 0.925 | −0.229 | 0.773 |

^a^No-exogenous models used influenza incidence history alone. Single-source models included digital prescription rate only. Multisource models included the variables specified in the scenario name (digital prescription rate, online search index, temperature, and, where applicable, NO₂). The multisource scenario without NO₂ was included as a sensitivity analysis to evaluate the incremental contribution of NO₂. Persistence carried the most recently observed influenza incidence forward to all horizons.

^b^Averaged over horizons of 1 to 14 days. Within each scenario, models are ordered by mean *R*².

ARIMA: autoregressive integrated moving average; EBM: explainable boosting machine; LASSO: least absolute shrinkage and selection operator; RF: random forest; RMSE: root mean square error; SARIMA: seasonal ARIMA.

**Figure S1.** Time-series heatmap of influenza incidence and the 13 candidate indicators, January 1, 2022, to December 31, 2024 (1096 days). Each cell is the daily value standardized within variable (z score): red denotes values above and blue denotes values below that variable’s own mean. Values were clipped at ±3 SD. Standardization was used for display only and did not enter any analysis. Horizontal black lines separate the variable categories, from top to bottom: outcome (influenza incidence), digital prescription data, online search index, human mobility, air quality, and meteorological conditions. SD: standard deviation.

**Figure S2.** Raw (unprewhitened) cross-correlation functions between each of the 13 candidate indicators and influenza incidence. Each panel (A-M) shows 1 indicator. Cross-correlation functions (CCFs) were computed as ccf(indicator, influenza incidence), with negative lags indicating that the indicator leads influenza incidence. Dashed lines denote descriptive nominal 95% bounds; bars exceeding these bounds are red, and the remaining bars are gray.

**Figure S3.** Prewhitened cross-correlation functions between each of the 13 candidate indicators and influenza incidence. Each panel (A-M) shows 1 indicator. Cross-correlation functions (CCFs) were computed after Box-Jenkins prewhitening by applying an autoregressive integrated moving average filter identified for the indicator to both series; negative lags indicate that the indicator leads influenza incidence. Dashed lines denote the Bartlett large-lag significance bounds (*P*<.05); bars outside these bounds are red, and the remaining bars are gray.

**Figure S4.** Granger test results (−log10 *P*; predictor-to-incidence direction) across daily lag orders for all 13 candidate indicators. Each panel (A-M) shows 1 indicator. The dashed line represents the nominal threshold of *P*=.05 (−log₁₀*P*=1.30). Bars above this line indicate stronger unadjusted per-lag *P* values below .05 and should not be interpreted as confirmatory statistical significance.

**Figure S5.** Temporal consistency between the forecasted online search index and observed influenza incidence across horizons of 1 to 14 days. Each panel (A-N) shows 1 horizon. The red line represents the online search index forecast from a univariate seasonal autoregressive integrated moving average model, with the 95% prediction interval shaded; the blue line represents observed influenza incidence. The vertical dashed line marks the start of the 2024 out-of-sample period; both series were minimum-to-maximum normalized to (0, 1) for display, and the Pearson correlation coefficient annotated in each panel was calculated using raw values.

**Figure S6.** Temporal consistency between the forecasted temperature and observed influenza incidence across horizons of 1 to 14 days. Each panel (A-N) shows 1 horizon. The red line represents the temperature forecast from a univariate seasonal autoregressive integrated moving average model, with the 95% prediction interval shaded; the blue line represents observed influenza incidence. The vertical dashed line marks the start of the 2024 out-of-sample period; both series were minimum-to-maximum normalized to (0, 1) for display, and the Pearson correlation coefficient annotated in each panel was calculated using raw values.

**Figure S7.** Temporal consistency between the forecasted NO₂ and observed influenza incidence across horizons of 1 to 14 days. Each panel (A-N) shows 1 horizon. The red line represents the NO₂ forecast from a univariate seasonal autoregressive integrated moving average model, with the 95% prediction interval shaded; the blue line represents observed influenza incidence. The vertical dashed line marks the start of the 2024 out-of-sample period; both series were minimum-to-maximum normalized to (0, 1) for display, and the Pearson correlation coefficient annotated in each panel was calculated using raw values.
